# Designing a more efficient, effective and safe Medical Emergency Team (MET) service using data analysis

**DOI:** 10.1371/journal.pone.0188688

**Published:** 2017-12-27

**Authors:** Christoph Bergmeir, Irma Bilgrami, Christopher Bain, Geoffrey I. Webb, Judit Orosz, David Pilcher

**Affiliations:** 1 Faculty of Information Technology, Monash University, Clayton, Australia; 2 Intensive Care Specialist, Departments of Anaesthesia, Intensive Care and Pain Management, Western Health, Gordon Street, Footscray, Vic, Australia; 3 Department of Intensive Care Medicine, Commercial Road, The Alfred Hospital, Prahran, Vic, Australia; 4 The Australian and New Zealand Intensive Care (ANZIC)–Research Centre, School of Public Health and Preventive Medicine, Monash University, Prahran, Vic, Australia; University Hospital Jena, GERMANY

## Abstract

**Introduction:**

Hospitals have seen a rise in Medical Emergency Team (MET) reviews. We hypothesised that the commonest MET calls result in similar treatments. Our aim was to design a pre-emptive management algorithm that allowed direct institution of treatment to patients without having to wait for attendance of the MET team and to model its potential impact on MET call incidence and patient outcomes.

**Methods:**

Data was extracted for all MET calls from the hospital database. Association rule data mining techniques were used to identify the most common combinations of MET call causes, outcomes and therapies.

**Results:**

There were 13,656 MET calls during the 34-month study period in 7936 patients. The most common MET call was for hypotension [31%, (2459/7936)]. These MET calls were strongly associated with the immediate administration of intra-venous fluid (70% [1714/2459] v 13% [739/5477] p<0.001), unless the patient was located on a respiratory ward (adjusted OR 0.41 [95%CI 0.25–0.67] p<0.001), had a cardiac cause for admission (adjusted OR 0.61 [95%CI 0.50–0.75] p<0.001) or was under the care of the heart failure team (adjusted OR 0.29 [95%CI 0.19–0.42] p<0.001).

Modelling the effect of a pre-emptive management algorithm for immediate fluid administration without MET activation on data from a test period of 24 months following the study period, suggested it would lead to a 68.7% (2541/3697) reduction in MET calls for hypotension and a 19.6% (2541/12938) reduction in total METs without adverse effects on patients.

**Conclusion:**

Routinely collected data and analytic techniques can be used to develop a pre-emptive management algorithm to administer intravenous fluid therapy to a specific group of hypotensive patients without the need to initiate a MET call. This could both lead to earlier treatment for the patient and less total MET calls.

## Introduction

Over the last two decades, Rapid Response Systems and Medical Emergency Teams (METs) have become widely adopted in hospitals across Australia and New Zealand [1,2]. It has become an expectation and a minimum standard of care that such systems detect and respond to physiological deterioration in a timely fashion [3,4]. The Australian Commission on Safety and Quality in Health Care (ACSQHC) has supported this endeavour and released a National Consensus Statement on management of the deteriorating patient [5]. Recent data suggests an increasing number of MET reviews and this number varies from 1.35/ 1000 admissions to 71.3/1000 admissions [6]. The reasons behind the increase and the variability are unclear. Local hospital culture and resource availability may play a role [7,8]. Only a limited number of hospitals receive funding dedicated to the MET [8]. This may have implications not only for resource allocation but also individual patient care.

The traditional design of a Rapid Response System has four components. An afferent arm or the event detection arm that triggers the response; an efferent arm or the responder arm (the MET); an administrative structure; and a quality improvement arm [9]. Given the rising number of rapid response calls, this ‘blanket’ efferent and afferent response to every trigger event may not be a sustainable model for the future.

We hypothesised that the commonest MET triggers resulted in similar treatments and that it might be possible to identify a cohort of patients in whom treatment could be directly initiated without having to await attendance of the MET. This might increase the efficiency of the MET system, through a total reduction in MET numbers and earlier administration of appropriate treatment to patients. Our aim was to develop a ‘pre-emptive management algorithm’ using available institutional data, for immediate administration of therapy at the most common MET calls and to model its potential impact on MET call incidence and patient outcomes, prior to implementation into real world practice.

Because large datasets of information are commonly now available in healthcare [10], advanced exploratory data analysis techniques that can identify potentially useful patterns in data are of ever greater value. Statistically sound association rule techniques systematically explore complex interaction in data while using multiple testing corrections to ensure statistical validity [11]. In consequence they are ideally suited to identifying the most common combinations of MET call causes, outcomes and therapies, allowing identification of combinations of variables that are useful in practice to incorporate into an algorithm.

## Methods

### Ethics

The Alfred Health Human Ethics Committee approved the study and provided a waiver for informed consent (Project 580/14).

### Setting

The Alfred hospital is a tertiary referral centre in Melbourne, Australia. It provides a state service for trauma, burns, cystic fibrosis, heart, lung and bone marrow transplant, extracorporeal cardiac and respiratory support, hyperbaric medicine and infectious diseases including HIV. The hospital admits more than 35,000 adult patients a year and has more than 3000 admissions to the Intensive Care Unit (ICU) per year.

The hospital has a mature MET service, established in early 2000s. The MET service is a 24-hour service, led by the ICU senior registrar and an Intensive Care trained nurse. The on call medical registrar and a member of the home team also attend. The MET responds to more than 6000 reviews every year. MET calls are mandatory if a patient meets pre-specified criteria (see supplementary material–S1 Table).

At the end of every MET call, the reason for the call, its location, therapy given and patient outcome are entered into a hospital wide MET database. The patient’s vital signs at the time of MET call are recorded in the patient’s records but not entered into the database.

### Study design

We conducted a retrospective observational study and extracted data for all MET calls between March 2012 (when the complete electronic MET database was implemented) and December 2016. Data from March 2012 to December 2014 was used to develop an algorithm which was subsequently validated on data from January 2015 to December 2016.

Data were extracted from the “REASON Discovery Platform” [12], the hospital’s corporate data platform which routinely aggregated, on a nightly basis, information from the MET database, demographics, pathology, admitting unit, admission/discharge dates from administrative databases and diagnostic coding data. The following data was collected for every MET call: timing of activation, the clinical trigger for the call and any therapies administered. We also measured patient outcomes post MET call including transfer to ICU or another high acuity area, repeat MET calls, length of stay in hospital and in-hospital mortality.

### Statistical analysis and association rule mining techniques

Association rule data mining techniques, implemented in the software Magnum Opus**®**, were used to search for patterns in the dataset and to identify the most common combinations of MET call causes, outcomes and therapies for inclusion in the ‘pre-emptive management algorithm’. Classical univariate pair-wise testing and multivariable logistic regression tests were then employed to check the validity of the findings from the association rule data mining and to test the potential impact of the algorithm.

Magnum Opus**®** performs statistically sound association rule mining, by combining k-optimal association discovery techniques [13], to find the k most interesting associations according to a defined criterion, such as lift, leverage, strength, support, or coverage. It achieves efficiency in the search by using the OPUS search method [14], and incorporates facilities for filtering the discovered associations to discard those that are likely to be spurious [11]. Magnum Opus® is available for use at [15], an open source implementation of OPUS is available at [16]. This advanced exploratory data analysis technique considers all possible correlations between combinations of up to three variables with a target. It uses the Bonferroni correction to ensure strict control over familywise error despite the combinatorial explosion in the number of tests performed. Interactions of interest are identified by measuring the frequency with which combinations of values occurred jointly, relative to the frequency that would be expected were the relevant combination of values statistically independent of the target.

Following identification of ‘candidate combinations’ of MET calls, outcomes and therapies using the above techniques, univariate statistical pair-wise testing without adjustment for multiple testing was employed using Student T-, Chi-square, Fisher’s exact or Wilcoxon rank sum tests as appropriate depending on the distribution of the data. Multivariable logistic regression in the R programming language [17], function *glm*, from the R stats package was used with associations reported as odds ratios with 95% confidence interval.

Only the first MET call per patient was considered for initial analysis and development of the algorithm. Modelling the effect of the algorithm was performed on this dataset. The primary outcome considered was the modelled number of MET calls, with the algorithm in place. Other outcomes considered included: in-hospital mortality, mortality at the MET call, proportion of patients admitted to ICU and length of stay in hospital.

## Results

The initial study period (March 2012 to December 2014), in the following also called the training period, was used for exploratory data analysis and to develop the algorithm. The results in the following are results from this period. During the training period, there were a total of 13,656 MET calls in 7936 patients. This equated to a MET dose of 144 METs per 1000 hospital admissions. MET call activity increased during the study period, with yearly mean daily counts of 11.0, 13.8, and 14.5 MET calls respectively, for the years 2012, 2013, and 2014. Frequencies of calling criteria are shown in Table 1. Of the 7936 patients, 7027 (88.5%) had a single calling criterion for MET activation.

**Table 1:** MET activations in the training period. Data includes only the first MET call per patient. MET = Medical Emergency Team, CPR = Cardiopulmonary resuscitation.

Total number of patients with METs	7936
Patients with METS for single calling criterion—n (%)	7027 (89%)
Patients with METS for multiple calling criteria- n (%)	909 (11%)
**Criteria breakdown**	
** MET single calling criterion—n (%)**	
Systolic blood pressure ≤ 90 mmHg	2459 (30.9%)
Heart rate ≥ 140 beats/min	1085 (13.7%)
Decrease in Glasgow Coma Score	962 (12.1%)
Oxygen saturations ≤ 90%	618 (7.8%)
Respiratory rate ≥ 36 breaths/min	429 (5.4%)
Systolic blood pressure ≥ 200 mmHg	359 (4.5%)
Serious concern	299 (3.8%)
Heart rate ≤ 40 beats/min	291 (3.7%)
Pain	252 (3.2%)
Seizures	116 (1.5%)
Cardiorespiratory arrest requiring CPR	76 (1.0%)
Respiratory rate ≤ 6 breaths/min	48 (0.6%)
Uncontrolled bleeding	33 (0.4%)
** MET multiple calling criteria—n (%)**	
Systolic blood pressure ≤ 90 mmHg + Decrease in Glasgow Coma Score	52 (0.7%)
Systolic blood pressure ≤ 90 mmHg + Heart rate ≤ 40 beats/min	45(0.6%)
Systolic blood pressure ≤ 90 mmHg + Heart rate ≥ 140 beats/min	43(0.5%)
Systolic blood pressure ≤ 90 mmHg + Oxygen saturations ≤ 90%	36(0.5%)
Systolic blood pressure ≤ 90 mmHg + Respiratory rate ≥ 36 breaths/min	11 (0.1%)
Systolic blood pressure ≤ 90 mmHg + Other	80 (1.0%)
Other	642 (8.1%)

### Identification of target group for algorithm development: The commonest MET call & therapy combination

Hypotension, defined as systolic blood pressure <90mmHg, was the most common sole MET call criterion (30.9%, 2459/7936). Hypotension was also present in 267 out of 909 cases with a combination of calling criteria, which led to activation of the MET (29.3%, 267/909) (Table 1)

Association rule data mining identified a MET call for hypotension and administration of intravenous fluid as the single most common combination of calling criterion and therapy in the training period. Compared to MET calls for other reasons, patients who had MET calls for hypotension were more commonly given intra-venous fluid therapy (70% [1714/2459] v 13% [739/5477] p<0.001), more commonly managed on the ward (94% [2316/2459] v 85% [4647/5477] p<0.003) and had fewer multiple METs (35% [867/2459] vs. 38% [2103/ 5477] p<0.008), were less likely to go to ICU (2% [52/ 2459] vs. 6% [311/5477] p<0.001) and had a lower mortality (5% [133/2459] vs. 11% [620/5477] p<0.001) than those who had MET calls for other reasons. Comparison of patients who received a MET review for hypotension to patients who received a MET review for other criteria is shown in S2 Table of the supplementary material.

### Development of the pre-emptive management algorithm for patients with MET calls for hypotension

Having identified the most common therapy given to hypotensive patients (intravenous fluid), the characteristics and outcomes of patients who received this therapy at a MET call were compared to those who were not given fluid (Table 2), in the training period. Patients given fluid were older, more commonly under the care of general surgical, trauma or orthopaedic units and wards, less commonly had primary cardiac diagnoses, and more commonly had more interventions performed at the MET call but remained on the ward with equal frequency compared to those where fluid was not administered. The only difference in outcomes between the two groups, was that patients who were given fluid had a higher number of multiple METs (38% [649/1714] vs. 29% [218/745] p<0.001).

**Table 2:** Comparison of patients who were given intravenous fluid therapy to those not given fluid therapy, at medical emergency team (MET) calls for hypotension, in the training period. Due to space constraints, for clinical units, MET call locations, and interventions, only results with (p < 0.05) are reported. mmol/L = Millimole per litre, micromol/L = micromoles per litre, g/dL = Grams per decilitre.

	All Patients (n = 2459)	Patients given fluid (n = 1714)	Patients not given fluid (n = 745)	p value
**Patient characteristics**				
Median age (Interquartile range)-years	66 (52–78)	64 (17–97)	61 (19–29)	0.004
Male gender n (%)	1184 (48%)	374 (53%)	49% (810)	0.12
Unknown gender n (%)	108 (4%)			
Hospital length of stay—median (Interquartile range) days	9 [4–17]	9 [4–17]	8 [4–16]	0.16
**Clinical units n (%)**				<0.01
Orthopaedics	279 (11%)	223 (13%)	56 (8%)	
Breast/endocrine surgery	42 (1.7%)	37 (2%)	5 (1%)	
Burns	40 (2%)	35 (2%)	5 (1%)	
Colorectal surgery	84 (3%)	73 (4%)	11 (1%)	
Plastics	65 (3%)	53 (3%)	12(2%)	
Urology	42 (2%)	38 (2%)	4 (1%)	
Heart failure	125 (5%)	46 (3%)	79 (11%)	
Psychiatry	22 (1%)	6 (0%)	16 (2%)	
General respiratory	43 (2%)	24 (1%)	19 (3%)	
Cystic Fibrosis	12 (0%)	5 (0%)	7 (1%)	
Lung transplantation	33 (1%)	16 (1%)	17 (2%)	
Neurology	37 (2%)	17 (1%)	20(3%)	
**MET call location n (%)**				<0.001
General surgery / burns ward	164 (7%)	138 (8%)	26 (3%)	
Trauma / orthopaedics ward	259 (11%)	205 (12%)	54 (7%)	
Trauma / neurosurgery ward	127 (5%)	101 (6%)	26 (3%)	
General surgery ward	157 (6%)	125 (7%)	32 (4%)	
Cardiology / Cardiothoracic ward	488 (20%)	295 (17%)	193 (26%)	
Psychiatry area	22 (1%)	6 (0%)	16 (2%)	
Respiratory ward	67 (3%)	35 (2%)	32(4%)	
**Primary Diagnoses (ICD 10 coding on admission to hospital) n (%)**				<0.001
(Circulatory / cardiac	562 (23%)	330 (19.3%)	232 (31.1%)	
Respiratory	226 (9%)	149 (8.7%)	77 (10.3%)	
Neurological	159 (6%)	110 (6.4%)	49 (6.6%)	
Hepatobiliary	77 (3%)	47 (2.7%)	30 (4%)	
Ear, nose, mouth & throat	37 (2%)	25 (1.5%)	12 (1.6%)	
Psychiatric	27 (1%)	10 (0.6%)	17 (2.3%)	
Musculoskeletal	380 (15%)	289 (16.9%)	91 (12.2%)	
Gastro-enterology	266 (11%)	210 (12.3%)	56 (7.5%)	
Infectious diseases	131 (5%)	101 (5.9%)	30 (4%)	
Renal & urology	124 (5%)	97 (5.7%)	27 (3.6%)	
Myeloproliferative diseases	92 (4%)	65 (3.8%)	27 (3.6%)	
Poisonings & toxicology	88 (4%)	70 (4.1%)	18 (2.4%)	
Endocrine & metabolic	57 (2%)	46 (2.7%)	11 (1.5%)	
Skin & breast	52 (2%)	38 (2.2%)	14 (1.9%)	
Other	40 (2%)	27 (1.6%)	13 (1.7%)	
Burns	38 (2%)	34 (2%)	4 (0.5%)	
**Haematology**	36 (1%)	26 (1.5%)	10 (1.3%)	
Alcohol/drug related	5 (0%)	2 (0.1%)	3 (0.4%)	
**Laboratory data at hospital admission–median (Interquartile range)**				
Urea (mmol/L)	6.6 [4.5–10.6]	6.6 [4.6–10.5]	6.45 [4.5–10.6]	0.64
Creatinine (micromol/L)	77 [63–114]	77 [63–113]	77 [63–117]	0.87
Haemoglobin (g/dL)	119 [105–132]	118 [103–130]	121.5 [108.5–133.5]	0.047
White cell count (x10^9/L)	7.9 [5.76–11]	8 [5.8–11.2]	7.8 [5.7–10.7]	0.21
**Laboratory data at MET call–median (Interquartile range)**				
Urea (mmol/L)	7 [4.7–11]	7 [4.6–11]	7.1 [4.7–10.9]	0.63
Creatinine (micromol/L)	78 [63–120]	78 [63–120]	79 [62–120]	0.76
Haemoglobin (g/dL)	104 [93–118]	105 [93–117]	102 [89–121.75]	0.46
White cell count (x10^9/L)	8.12 [6–11.3]	8.5 [6.1–11.8]	7.7 [5.9–10.1]	0.016
**Interventions**				
Advice or consult only	563 (23%)	51 (3%)	512 (69%)	<0.001
Blood test	873 (36%)	751 (44%)	122 (16%)	<0.001
Electrocardiogram	1166 (47%)	948 (55%)	218 (29%)	<0.001
Oxygen up to 6l/min	181 (7%)	156 (9%)	25 (3%)	<0.001
Arterial Blood Gas	176 (7%)	145 (8%)	31 (4%)	0.001
X-ray	123 (5%)	96 (6%)	27 (4%)	0.039
**MET Outcomes n (%)**				
Transferred to Intensive Care Unit	52 (2%)	41 (2%)	11(1%)	0.15
Died at the MET call	0 (0%)	0 (0%)	0 (0%)	1.00
In-hospital mortality	133 (5%)	98 (6%)	35(5%)	0.30
Multiple MET calls	867 (35%)	649 (38%)	218(29%)	<0.001
Stayed in the ward	2316 (94%)	1615 (94.2%)	701 (94.1%)	0.98
Palliative care initiated	9 (0%)	6 (0%)	3(0.4%)	1.00

Association rule data mining, together with a subsequent multivariate logistic regression confirmed that the most important correlations were seen if the patient was located on a respiratory ward (adjusted OR 0.41 [95%CI 0.25–0.67] p<0.001), had a cardiac cause for admission (adjusted OR 0.61 [95%CI 0.50–0.75] p<0.001) or was under the care of the heart failure team (adjusted OR 0.29 [95%CI 0.19–0.42] p<0.001), in which case the patient was less likely to receive intravenous fluid.

**Fig 1:**
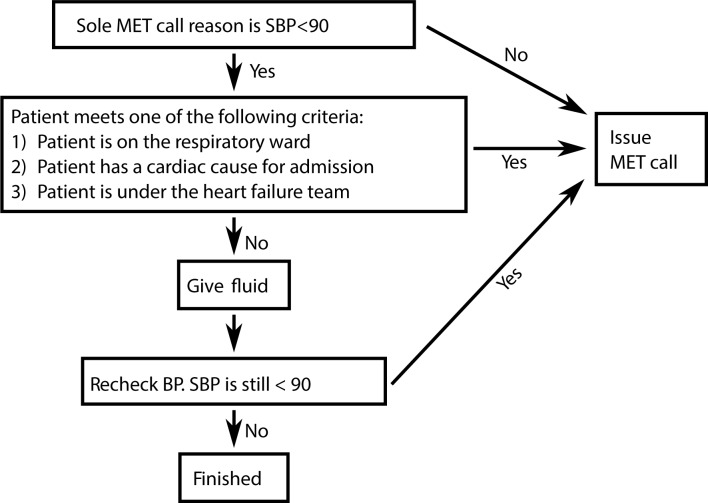
Flow chart of the proposed algorithm.

Based on the above findings from the training period, an algorithm for direct nurse initiated administration of intravenous fluid was developed and its potential impact on MET call numbers and patients’ outcomes was modelled. The algorithm is shown in Fig 1. Unless on the respiratory ward, admitted with a cardiac diagnosis or under the care of the heart failure team, any patient who had systolic hypotension < 90mmHg was to receive intravenous fluid without need for a MET call. In all other cases, a MET was to be called.

#### Modelling the impact of the pre-emptive management algorithm

We subsequently assessed the impact of the algorithm using a separate test dataset, comprised of 12,938 MET calls in 7106 patients from the 24 months period of January 2015 to December 2016. All the results in the following are results for this test period.

#### Assessment of primary outcome

If the algorithm had been applied, there would have been a 68.7% (2541/3697) reduction in MET calls for hypotension and a 19.6% (2541/12938) reduction in total METs (Table 3) in the test period. This would also have resulted in 50.0% (1045/2091) patients who had a MET call for hypotension, receiving the intravenous fluids without actually having to activate a MET review (‘true positives’). All other patients would have continued to receive a MET review.

**Table 3:** Modelled estimated effect of algorithm for immediate administration of intravenous fluid to patients with systolic blood pressure (SBP) < 90mmHg, on medical emergency team (MET) calls in the test period.

		Without Algorithm	With Algorithm
**Number of MET calls (total)**		12938	10397 (19.6% reduction)
**Number of patients with one or more MET call**		7106	5658 (20.4% reduction)
**Number of patients with first MET call for SBP <90 mmHg**		2091	643 (69.2% reduction)
**Number of MET calls for SBP <90 mmHg**		3697	1156 (68.7% reduction)
**Fluid administration in patients with hypotension at MET–n (%)**			
	Given intravenous fluid	1365 (65.3%)	n/a
	Not given intravenous fluid	726 (34.7%)	n/a
	Predicted to get intravenous fluid	n/a	1448 (69.2%)
	Predicted not to get intravenous fluid	n/a	643 (30.8%)
	Predicted not to get intravenous fluid and did not receive it (true negatives)	n/a	323 (15.4%)
	Predicted not to get intravenous fluid and did receive it (false negatives)	n/a	320 (15.3%)
	Predicted to get intravenous fluid and did receive it (true positives)	n/a	1045 (50.0%)
	Predicted to get intravenous fluid and did not receive it (false positives)	n/a	403 (19.3%)

### Assessment of outcomes in patients in whom the algorithm predicted ‘inadvertent fluid therapy’

The algorithm would have resulted in intravenous fluid administration to 19.3% (403/2091) of patients who did not actually receive it (‘false positives’ or ‘inadvertent fluid therapy’)(Table 3), in the test period. These patients (the ‘false positives’) appeared to be a ‘lower risk’ group, when compared to those who both received intravenous fluids and the algorithm predicted fluid administration. They had lower mortality and in 57.8% of these patients, the MET provided advice only and did not order any investigations or prescribe a therapy (Table 4).

**Table 4:** Comparison of interventions and outcomes of hypotensive patients who did receive intravenous fluid and were predicted to do so by the algorithm (‘true positives’), vs. those who did not receive intravenous fluid but would have been predicted to do so (‘false positives’), in the test period. ‘True positives’ represent those with MET calls for SBP <90 mmHg, predicted to get intravenous fluid who did receive it. ‘False positives’ represent those with MET calls for SBP <90 mmHg, predicted to get intravenous fluid who did not receive it. The p values are for comparison between True and False positive cases. MET = Medical Emergency Team, ECG = Electrocardiogram, ABG = Arterial Blood Gas.

		All patients with a MET call for SBP <90 mmHg (n = 2091)	True positive cases (n = 1045)	False positive cases (n = 403)	p value
**Interventions at MET call**					
**Advice or consult only**		402 (19.2%)	0 (0%)	233 (57.8%)	<0.001
**Investigations**					
	ECG	904 (43.2%)	511 (48.9%)	113 (28.0%)	<0.001
	ABG	199 (9.5%)	114 (10.9%)	20 (5.0%)	0.001
	Blood test	577 (27.6%)	376 (36.0%)	64 (15.9%)	<0.001
	X-ray	99 (4.7%)	57 (5.5%)	13 (3.2%)	0.10
**Therapies (other than iv fluids)**					
	Oxygen	105 (5.0%)	57 (5.5%)	13 (3.2%)	0.10
	Medication change	238 (11.4%)	119 (11.4%)	29 (7.2%)	0.033
	Non-invasive ventilation	5 (0.2%)	0 (0.0%)	2 (0.5%)	0.078
**Outcomes at MET call**					
	Deceased	183 (8.8%)	106 (10.1%)	25 (6.2%)	0.031
	Transferred to ICU	77 (3.7%)	50 (4.8%)	13 (3.2%)	0.25
	Multiple MET calls	1385 (66.2%)	717 (68.6%)	256 (63.5%)	0.43
	Any one of above outcomes	1409 (67.4%)	730 (69.9%)	259 (64.3%)	0.38

## Discussion

### Summary of findings

In this study of 7936 patients at a tertiary centre over a 34-month period (training period), the most common combination of MET call and therapy was observed in patients who received intravenous fluid at a MET call for a systolic blood pressure < 90mmHg. A combination of data association mining and classical statistical techniques allowed the development and assessment of a pre-emptive management algorithm for immediate management of these patients without having to initiate a MET call. Modelling the effect of this algorithm for immediate administration of intravenous fluid to hypotensive patients on an independent test period suggested that it would lead to a reduction in overall numbers of MET calls and if applied, it would have led to inadvertent intravenous therapy to a small proportion of low risk patients and was thus unlikely to result in adverse effects to patients.

### Comparison to previous studies

Literature describing common MET triggers is limited [6]. These have been described in various frequencies in single centre studies [18,19]. In our study, the most common trigger for a MET response was a systolic blood pressure <90mmHg. These patients were more likely to be managed on the ward and less likely to have multiple METs, an important finding as patients with repeat METs are known to be a higher risk group [20]. In hospital mortality of hypotensive patients in our study (5%) was lower than the range reported in literature (25%) [6]. The ICU admission rate (2%) was also lower than the reported rates in literature (10–25%). These outcomes suggest that this is an ideal group for application of a pre-emptive management algorithm.

Data describing therapies administered during MET calls is limited [19,21]. One study found oxygen therapy or ventilatory support as the most common intervention provided by MET [19]. We found MET reviews for hypotension were strongly associated with fluid administration unless the patient was on the respiratory ward, had a cardiac diagnosis or was admitted under the heart failure team. We also found that patients receiving fluid therapy at a MET call for hypotension had a higher number of multiple METs than those who did not receive fluid therapy. The relevance of this is unclear as all other patient outcomes were similar in both groups.

### Implications

We have shown that routinely collected hospital data can be used to develop a pre-emptive management algorithm, which might facilitate administration of therapy to a specific group of hypotensive patients. Modelling implementation of this algorithm suggested its introduction would be associated with a reduction in MET call numbers without compromising patient outcomes. In the face of rapid adoption of MET systems and an increasing workload [2] this demonstrates the potential systems redesign and safe resource reallocation.

Patients who receive a MET response are a high-risk group [6,7] and a delay in activating MET calls leads to higher mortality [22]. The traditional model of the MET system has a disadvantage [2,9] in that treatment can only be given once the effector response arrives at the patient’s bedside. Given the high MET dose in our hospital, there is a danger of delayed response times with the potential to cause harm. One of the potential advantages of implementing this pre-emptive management algorithm is to avoid delay.

### Strengths

We used a large, well-established database run by trained staff. The study period of 34 months allowed us to study established trends. We used association rule mining techniques to examine the data and results were consistent when the standard statistical techniques were utilised. The data mining techniques involved can be generalised to any large dataset. We have demonstrated that evolution and development of clinical systems may be achieved through analysis of data rather than relying solely on opinion, supporting a move towards evidence based practice.

The algorithm designed was simple and is directly applicable to our hospital setting which makes likelihood of successful implementation greater. Internal validity of our study was supported by biologically plausible findings such as the fact that cardiac and respiratory patients who had hypotension were less likely than others to receive intravenous fluid.

### Limitations

This was an observational study and the impact of unmeasured confounders (e.g. staffing levels, education, handover processes) is unknown. This work describes methodology for development of a management algorithm and cannot be considered definitive assessment of its applicability. This work is the first phase prior to assessment of such an algorithm in practice. The proposed algorithm has not been implemented or tested in a controlled trial or in clinical practice. Moreover, although the algorithm has been prospectively validated, and the principles of its development may be broadly relevant, since this is a single centre study, the applicability of the findings and the relevance of the algorithm to other hospitals and healthcare systems is unknown.

Furthermore, although our analysis suggested that patients who might be managed by our proposed algorithm (and would not have a MET call) were a 'low risk' group, a retrospective study such as this cannot fully address all potential safety concerns with its implementation. For instance, there might need to be a systolic blood pressure level albeit a lower value, at which a MET call would still have to be called.

Accuracy of data elements is unknown. Absolute values of systolic blood pressure were not recorded. Hence it is unknown if there are subgroups of patients with very low blood pressures which might be disadvantaged by our algorithm and failure to immediately activate a MET call. However, the relatively good outcomes in these patients suggested they were not a high-risk group overall. The incidence, quality and impact of clinical assessment of patients is unknown. However it is recognised that fluid assessment in the critically ill is challenging and the accuracy of physical assessment in determining fluid state has been questioned [23].

### Future work

Future studies should assess whether the same analytic techniques can be applied in other hospital settings to develop similar algorithms to improve MET systems. Locally, routine recording of absolute numerical values for physiological observations at MET calls is needed, and careful assessment of the algorithm in a clinical arena is required.

## Conclusion

Using routinely collected data and specific analytic techniques, it is possible to develop a pre-emptive management algorithm to administer intravenous fluid therapy to a specific group of hypotensive patients without the need to initiate a MET call. Application of such an algorithm is likely to be safe, result in a reduction in total MET calls, improved efficiency of the MET system and may lead to improved patient outcomes.

## Supporting information

S1 Table MET calling criteria. (PDF)

S2 Table Comparison of medical emergency team (MET) calls for hypotension to MET calls for other reasons in the training period. (PDF)

S3 Table Patient characteristics associated with no fluid administration, in the training period. (PDF)
