# Supplementary material for: Designing a more efficient, effective and safe Medical Emergency Team (MET) service using data analysis
Source: PLoS One. 2017 Dec 27;12(12):e0188688. doi: 10.1371/journal.pone.0188688 (PMC5744916; doi:10.1371/journal.pone.0188688)
Supplement: S1 Table — (PDF) [file pone.0188688.s001.pdf]

S1 Table: MET calling criteria.

Vital Sign	MET Call Criteria
Airway	Threatened
Respiratory rate	≤ 6 breaths/min OR ≥ 36 breaths/min
Oxygen saturations (on air or oxygen)	≤ 90%
Systolic blood pressure	≤ 90 mmHg OR ≥ 200 mmHg
Heart rate	≤ 40 beats/min OR ≥ 140 beats/min
Conscious state	Any unexpected decrease in level of consciousness
	Fall in GCS >2 points
	Seizures
Other	Serious concern
	Uncontrolled pain

'MET' Medical Emergency Team
'GCS' Glasgow Coma Score
