# Supplementary material for: Designing a more efficient, effective and safe Medical Emergency Team (MET) service using data analysis
Source: PLoS One. 2017 Dec 27;12(12):e0188688. doi: 10.1371/journal.pone.0188688 (PMC5744916; doi:10.1371/journal.pone.0188688)
Supplement: S2 Table — (PDF) [file pone.0188688.s002.pdf]

S2 Table: Comparison of Medical Emergency Team (MET) calls for hypotension to MET calls for other reasons in the training period.

	**All patients (n= 7936)**	**Number of patients with METs for hypotension (n=2459)**	**Number of patients with METS for other reasons (n=5477)**	**p value**
**Patient characteristics**				
Median age [Interquartile range]- years	**68 [53-80]**	66 [52-78]	69 [54-81]	<0.001
Male gender-n (%)<br>Unknown	4093 (54%)<br>375 (5%)	1184 (50%)	2909 (56%)	<0.001
Hospital length of stay - median (Interquartile range) days	9.5 [5-18]	9 [4-17]	10 [5-19]	<0.001
**Clinical Unit-n (%)**				<0.001
Heart Failure	246 (3%)	125 (5%)	121 (2%)	
Gastroenterology	221 (3%)	109 (4%)	112 (2%)	
Renal	277 (3%)	113 (5%)	164 (3%)	
Infectious disease	224 (3%)	105 (4%)	119 (2%)	
Colorectal surgery	187 (2%)	84 (3%)	103 (2%)	
Orthopaedics	582 (7%)	279 (11%)	303 (6%)	
Plastics	132 (2%)	65 (3%)	67 (1%)	
Breast/ endocrine surgery	103 (1%)	42 (2%)	61 (1%)	
Burns	87 (1%)	40 (2%)	47 (1%)	
General Medicine	1627 (21%)	414 (17%)	1213 (22%)	
Lung transplantation	213 (3%)	33 (1%)	180 (3%)	
Stroke	239 (3%)	14 (1%)	225 (4%)	
General respiratory	217 (3%)	43 (2%)	174 (3%)	
Asthma/allergy	19 (0%)	1 (0%)	18 (0%)	
Psychiatry	101 (1%)	22 (1%)	79 (1%)	
Cardiothoracic Surgery	510 (6%)	113 (5%)	397 (7%)	
Neurosurgery	378 (5%)	54 (2%)	324 (6%)	
Trauma	399 (5%)	80 (3%)	319 (6%)	
**MET call location -n (%)**				<0.001
General medicine / Renal ward	193 (2%)	88 (4%)	105 (2%)	
General medicine 1	215 (3%)	111 (5%)	104 (2%)	
General surgery	394 (5%)	157 (6%)	237 (4%)	
Short stay surgical unit	264 (3%)	127 (5%)	137 (3%)	
Trauma / orthopaedics	736 (9%)	259 (11%)	477 (9%)	
General surgery / burns ward	447 (6%)	164 (7%)	283 (5%)	
General medicine 2	1560 (20%)	416 (17%)	1144 (21%)	
Respiratory	400 (5%)	67 (3%)	333 (6%)	
Trauma/ Neurosurgery	606 (8%)	127 (5%)	479 (9%)	
Psychiatry	109 (1%)	22 (1%)	87 (2%)	

**Diagnosis (ICD 10 coding on admission to hospital) - n (%)**				<0.001
Musculoskeletal	894 (11%)	380 (15.5%)	514 (9.4%)	
Gastroenterology	609 (8%)	266 (10.8%)	343 (6.3%)	
Infectious diseases	339 (4%)	131 (5.3%)	208 (3.8%)	
Renal & Urology	293 (4%)	124 (5%)	169 (3.1%)	
Myeloproliferative diseases	261 (3%)	92 (3.7%)	169 (3.1%)	
Hepatobiliary	233 (3%)	77 (3.1%)	156 (2.8%)	
Endocrine & metabolic	138 (2%)	57 (2.3%)	81 (1.5%)	
Skin & breast	136 (2%)	52 (2.1%)	84 (1.5%)	
Haematology	100 (1%)	36 (1.5%)	64 (1.2%)	
Other	93 (1%)	40 (1.6%)	53 (1%)	
Burns	86 (1%)	38 (1.5%)	48 (0.9%)	
Poisonings & toxicology	298 (4%)	88 (3.6%)	210 (3.8%)	
Circulatory/ cardiac	1905 (24%)	562 (22.9%)	1343 (24.5%)	
Respiratory	1123 (14%)	226 (9.2%)	897 (16.4%)	
Neurological	954 (12%)	159 (6.5%)	795 (14.5%)	
Alcohol/ drug related	22 (0%)	5 (0.2%)	17 (0.3%)	
Ear, nose, mouth & throat	126 (2%)	37 (1.5%)	89 (1.6%)	
Psychiatric	120 (2%)	27 (1.1%)	93 (1.7%)	
NA	206 (3%)	62 (2.5%)	144 (2.6%)	
**Laboratory data at hospital admission – median [Interquartile range]**				
**Urea (mmol/L)**	6.7 [4.7-10.2]	6.6 [4.5-10.6]	6.7 [4.7-10]	0.83
**Creatinine (micromol/L)**	77 [64-109]	77 [63-114]	77 [64-107]	0.57
**Haemoglobin (g/dL)**	121 [107-135]	119 [105-132]	123 [108-137]	<0.001
**White cell count (x10^9/L)**	8.6 [6.3-11.6]	7.9 [5.76-11]	8.8 [6.6-11.8]	<0.001
**Laboratory data at MET call – median (Interquartile range)**				
**Urea (mmol/L)**	7.1 [4.8-10.7]	7 [4.7-11]	7.1 [4.9-10.7]	0.50
**Creatinine (micromol/L)**	76 [62-112]	78 [63-120]	75 [62-108]	0.006
**Haemoglobin (g/dL)**	105 [92-120]	104 [93-118]	105 [92-120]	0.31
**White cell count (x10^9/L)**	8.9 [6.6-12]	8.12 [6-11.3]	9.2 [6.9-12.3]	<0.001
**MET Outcomes %(n)**				
Transferred to Intensive Care Unit	363 (5%)	52 (2%)	311 (6%)	<0.001
Died at MET call	42 (1%)	0 (0%)	42 (1%)	<0.001
In-hospital Mortality	753 (9%)	133 (5%)	620 (11%)	<0.001

Multiple MET calls	2970 (37%)	867 (35%)	2103(38%)	0.008
Stayed in the Ward	6963 (88%)	2316 (94.2%)	4647 (84.8%)	0.003
Palliative care initiated	90 (1%)	9 (0.4%)	81 (1.5%)	<0.001

Due to space constraints, for clinical units and MET call locations, only results with ($p < 0.05$) are reported.
"*mmol/L* " Millimole per litre
"*micromol/L* " micromoles per litre
"*g/dL* " Grams per decilitre
