# Supplementary material for: Designing a more efficient, effective and safe Medical Emergency Team (MET) service using data analysis
Source: PLoS One. 2017 Dec 27;12(12):e0188688. doi: 10.1371/journal.pone.0188688 (PMC5744916; doi:10.1371/journal.pone.0188688)
Supplement: S3 Table — (PDF) [file pone.0188688.s003.pdf]

S3 Table: Patient characteristics associated with no fluid administration, in the training period.

	*Statistical analysis	Association rule mining
**Clinical unit**	Heart Failure, Psychiatry, Neurology	Heart failure
**Wards**	Cardiology, Psychiatry, Respiratory ward	Respiratory ward
**Admission diagnosis**	Multiple	Cardiac

* Associations with p value <0.002 are used. This is comparable to the Bonferroni correction applied in the association rule mining.
